# Supplementary material for: Epidemiology of hepatitis B and C virus infections among patients who booked for surgical procedures at Felegehiwot referral hospital, Northwest Ethiopia
Source: PLoS One. 2020 Jun 17;15(6):e0234822. doi: 10.1371/journal.pone.0234822 (PMC7299365; doi:10.1371/journal.pone.0234822)
Supplement: S1 Table — (PDF) [file pone.0234822.s001.pdf]

Annex: Questionnaire to assess the risk of getting HBV and HCV infections among pre surgery patients.

Sr.No.	Questions	Response category
**A**	**Socio-demographic questions**	
11	Age in years	___________
12	Sex	1). Male 2). Female
13	Religion	1). Orthodox 2). Muslim 3). Catholic<br>4). Protestant 5). Other____________
14	Residence	1). Kebele 1.) Urban, 2. Rural)<br>2). District (rural, urban)
15	Education level	1). No formal education<br>2). Primary school completed (1-8)<br>3). Secondary school completed (9-12)<br>4). Above secondary (College, University)
16	Marital status	1). Single 2). Married (in union)<br>3). Widowed 4). divorced
17	Occupation	1). Farmer 2). Employed<br>3). Non-employed
18	HIV status	1) Positive 2). Negative<br>3). Unknown
**B**	**Risk assessment questions**	
19	Have you ever heard about the disease hepatitis?	1). YES 2). No
20	Do you have experience of multiple sexual intercourses?	1). Yes 2). No
21	If yes for Q 20, with whom you did sex?	1). Girlfriends 2). Commercial sex workers<br>3). Wives 4). No status is known
22	When did you do practice multiple practices?	___________________
23	Do you have experience of sharing sharp materials (needle, blade, pin, et.c) with others in the family, relatives or neighbors?	1). Yes 2). No

24	If yes for Q23, did you get injection or cutting with those shared sharp materials?	1). Yes 2). No 3). Do not remember
25	Did you get blood transfusion from others in your life?	1). Yes 2). No
26	If yes for Q25, when you did it?	________________________
27	Did you practice tooth extraction due to illness?	1). Yes 2). No
28	If yes for Q27, where?	1). At home 2). Traditional practices<br>3). Clinics 4). Health center<br>5). Hospital 6). Other_________
29	Were you admitted at health facilities before?	1). Yes 2). No
30	Did you do surgical procedures (minor or major surgery) before?	1). Yes 2). No
31	Did you come across with others' blood contact with bear hand in your life time?	1). Yes 2). No
32	Did you get needle stick anywhere in your life time?	1). Yes 2). No
